# Prevalence, risk factors and severity of symptoms of pelvic organ prolapse among Emirati women

**DOI:** 10.1186/s12894-015-0062-1

**Published:** 2015-07-07

**Authors:** Hassan M. Elbiss, Nawal Osman, Fayez T. Hammad

**Affiliations:** Department of Obstetrics and Gynaecology, College of Medicine and Health Sciences, United Arab Emirates University, Al Ain, United Arab Emirates; Department of Surgery, College of Medicine and Health Sciences, United Arab Emirates University, Al Ain, PO Box 17666, United Arab Emirates

**Keywords:** Genital organ prolapse, Prevalence, Risk factors, Emirati women

## Abstract

**Background:**

Similar to other Gulf countries, the society in United Arab Emirates is pro-natal with high parity and high prevalence of macrosomic babies. Therefore, it is possible to have a high prevalence of pelvic organ prolapse (POP). Thus, the aim of this study was to determine the prevalence of POP symptoms in one of the UAE cities.

**Methods:**

A cross-sectional study of all women who attended the three family development centres was conducted in Al-Ain from January 2010 to January 2011. Non-Emirati, pregnant and nulliparous women younger than 30 years were excluded.

**Results:**

Out of 482 women who met the inclusion criteria, 429 (89.0 %) agreed to fully participate in the study. 127 women (29.6 %) reported symptoms of POP (mean age: 38.2 years, range: 18–71).

Out of the 127 affected women, a dragging lump was felt occasionally in 68 %, sometimes in 19 %, most of times in 9 % and all the times in 4 %. 73 % of affected women experienced soreness in the vagina. Around one third had to insert their fingers in the vagina to either start or complete emptying of the bladder or to empty the bowel.

Using multivariate analysis, the independent risk factors were history of constipation, level of education, chronic chest disease, nature of occupation, birth weight and body mass index (Odds ratio; 95 % Confidence interval): (4.1; 2.3-7.3), (1.7; 1.2-2.3), (2.9; 1.6-5.5), (0.5; 0.4-0.8), (1.7; 1.1-2.5), (1.1; 1.0-1.1), respectively (P < 0.05 for all).

**Conclusion:**

Symptoms of POP are prevalent among Emirati women. Independent risk factors included history of chronic constipation and chest disease, level of education, job type, birth weight and body mass index. Additional healthcare campaigns are required to educate the public regarding these risk factors.

**Electronic supplementary material:**

The online version of this article (doi:10.1186/s12894-015-0062-1) contains supplementary material, which is available to authorized users.

## Brief summary

Symptoms of POP are common among Emirati women. Several risk factors were identified. Healthcare campaigns are required to educate the public regarding these risk factors.

## Background

Female pelvic floor organ prolapse (POP) is a relatively common condition which might result in bothering symptoms. The prevalence of POP and associated symptoms vary among different studies depending on the population studied and research methodology. On examination, 32-41 % of women were found to have some degree of POP [1, 2]. However, among women with POP, 3–8.3 % have prolapse-related symptoms [3–8].

POP is associated with several risk factors such as multiparity and macrosomia [2, 3, 9]. In this regards the communities in the Gulf countries including UAE are generally pro-natal and hence, women tend to have high parity and short inter-pregnancy intervals. Moreover, these countries also report a high prevalence of macrosomic neonates due to increased prevalence of gestational diabetes [10]. Therefore, the prevalence of POP in UAE may be higher than those reported from the West.

No study has been conducted previously to investigate the prevalence of POP and associated risk factors among women in this part of the world. Therefore, the aim of the present study was to evaluate the prevalence, risk factors and severity of POP symptoms among women in UAE.

## Methods

A cross-sectional study of all Emirati women attending all the three family development centres in Al Ain, UAE was conducted from January 2010 to January 2011. These centres are the main and the only governmental facilities in the city and are visited by large number of Emirati women with different ages and backgrounds. Non-Emirati, pregnant and nulliparous women younger than 30 years were excluded. Approval was obtained from the Research Ethical Committee at the College of Medicine and Health Sciences, UAE University. Written consent was obtained from all eligible participants.

The initial phase of the study included developing the required questionnaire for data collection (Additional file 1), testing it to suite our population’s attitudes to discuss issues related to POP symptoms. To achieve this, a pilot study was performed on 20 randomly-selected female staff working at the College of Medicine and Health Sciences, UAE University who met the selection criteria of the study. Subsequent modifications were made to the questionnaire based on the results of this pilot study and the questionnaire was then retested on the same group to ensure its suitability in identifying the issues to be addressed in the present study.

The second phase included interviewing all the eligible subjects in the family development centres face to face by well-trained healthcare providers. After explaining the survey to the eligible subjects, all women who agreed to participate were asked to sign a consent form. This was followed by administering the pre-tested questionnaire. The questionnaire consisted of items related to socio-demographic, obstetrics, medical and surgical history. The woman was then asked if she had a dragging lump coming down in the vagina, lump coming out of vagina or lump felt or seen outside vagina; the presence of any of these symptoms were considered to indicate the presence of POP in this study. This was followed by other questions to determine the severity of the condition, other vaginal symptoms and if the women had to insert her finger into the vagina to reduce the lump in order to be able to empty the bladder or bowel.

Data was analysed using SPSS version 19.0 (IBM, Armonk, NY, USA). The subjects were classified into two groups: those with and those without POP symptoms. Inter-group comparisons were performed initially using univariate analysis for all potential risk factors. Student-*t* test was performed for continuous variables and chi-square or Fisher exact tests for categorical variables. Multivariate direct binary logistic regression analysis was, then, performed on all variables which showed significance on univariate analysis, to determine independent risk factors for POP. A P value of <0.05 was considered statistically significant.

## Results

Out of 482 women approached and met the inclusion criteria, 429 (89.0 %) agreed to fully participate in the study. Out of these, there were 127 women (29.6 %) who reported symptoms of POP with a mean age of mean age of 38.2 years (range: 18–71). There was no significant difference in the age between the group with and the group without POP symptoms as shown in Table 1.

**Table 1:** Socio-demographic characteristics of women with and without POP symptoms. All variables are expressed as number and percentage from the total number of patients from the respective group (with or without POP symptoms) except age and body mass index which were expressed as mean ± standard error of the mean

	No POP Symptoms	POP Symptoms	P-value
	(*n* = 302)	(*n* = 127)	
Age (in years)	37.4 ± 0.6	38.2 ± 0.9	0.45
Body Mass Index (BMI)	28.2 ± 0.3	29.9 ± 0.5	0.004
Monthly income (AED)			
<5000	36 (12 %)	4 (3 %)	
5000-10000	161 (53 %)	55 (43 %)	0.07
>10000	105 (35 %)	70 (55 %)	
Education			
Illiterate	82 (27 %)	29 (23 %)	
Primary School	98 (32 %)	54 (43 %)	0.009
Secondary School	61 (20 %)	33 (26 %)	
University	61 (20 %)	11 (9 %)	
Occupation			
Housewife	198 (66 %)	91 (72 %)	
Office Job	32 (11 %)	19 (15 %)	0.036
Physical Job	72 (24 %)	17 (13 %)	
Marital Status			
Never married	11 (3 %)	2 (0.5 %)	
Married/ previously married	291 (68 %)	125 (29 %)	0.3

The socio-demographic characteristics are shown in Table 1. Several socio-demographic factors were significantly associated with POP symptoms. These included body mass index (BMI) (women with POP symptoms were more obese than those with no symptoms, *P* = 0.004). As shown in Table 1, it appears that women with university degree had a lower incidence of POP symptoms compared to those with lower degree of education or illiterate women (9 % of women with POP symptoms were university graduates compared to 20 % of those without POP symptoms) (*P* = 0.009).

There was a tendency for women with higher income to have more prevalence of POP symptoms compared to women with lower income although this did not reach statistical significance. The type of occupation also affected the prevalence of POP symptoms which was more prevalent among housewives and women with office jobs compared to those who had jobs which required physical effort (only 13 % of women with POP symptoms had physical jobs compared to 24 % of those without POP symptoms). Marital status, however, did not affect the prevalence of POP symptoms.

Table 2 summarizes the medical, surgical, and obstetrics data in relation to POP symptoms. Some medical diseases or conditions were significantly associated with POP symptoms. These included chronic chest disease, constipation and Diabetes Mellitus. History of smoking was not associated with an increased prevalence of POP symptoms.

**Table 2:** Medical and Obstetric history of women with and without POP symptoms. All variables are expressed as number and percentage from the total number of patients from the respective group (with or without POP symptoms) parity and maximum birth weight which were expressed as mean ± standard error of the mean

	No POP Symptoms	POP Symptoms	P-value
	(*n* = 302)	(*n* = 127)	
Chronic Chest disease	33 (11 %)	35 (28 %)	0.0001
Constipation	32 (11 %)	48 (38 %)	0.0001
Diabetes Mellitus	45 (15 %)	29 (23 %)	0.047
Smoking	25 (8 %)	12 (9 %)	0.7
Parity	4.6 ± 0.2	4.5 ± 0.3	0.7
Previous instrumental delivery	23 (8 %)	20 (16 %)	0.01
Previous emergency LSCS	49 (16 %)	20 (16 %)	0.6
Previous elective LSCS	13 (4 %)	4 (3 %)	0.3
Maximum birth weight	3.3 ± 0.04	3.49 ± 0.06	0.005
History of urinary incontinence	94 (31 %)	88 (69 %)	0.0001
Previous surgery for urinary incontinence	11 (4 %)	11 (9 %)	0.03
Previous surgery for POP	2 (1 %)	20 (16 %)	0.0001

Some of the features in the previous obstetric history affected the prevalence of POP symptoms whereas others did not. For instance, parity and the previous history of elective or emergency caesarean sections did not affect whereas the history of previous instrumental delivery and high birth weight significantly increased the prevalence of POP symptoms. As might be expected, women with POP symptoms had a significantly higher incidence of urinary incontinence and previous history of POP or urinary incontinence surgery.

Despite the fact that several factors were significantly associated with the symptoms of POP on univariate analysis, multivariate logistic regression of these factors (BMI, level of education, nature of occupation, history of chronic chest disease, constipation, diabetes mellitus, previous instrumental delivery, maximum birth weight, history of urinary incontinence and previous surgery for urinary incontinence) revealed that there were only few independent risk factors. These include the history of constipation, level of education, chronic chest disease, nature of occupation, maximum birth weight and BMI (Table 3).

**Table 3:** The independent risk factors for POP symptoms in women using multivariate logistic regression analysis

	Odds ratio	95 % CI	*P* value
Constipation	4.1	2.3-7.3	0.0001
Education	1.7	1.2-2.3	0.001
Chronic Chest Disease	2.9	1.6-5.5	0.001
Occupation	0.5	0.4-0.8	0.002
Maximum Birth Weight	1.7	1.1-2.5	0.016
Body Mass Index	1.1	1.0-1.1	0.046

In regard to the severity of POP symptoms, out of the 127 patients with symptoms of POP, the dragging lump was felt occasionally in 68 % of the women, sometimes in 19 %, most of times in 9 % and all the times in 4 %. In addition to the dragging sensation, the majority of affected women had soreness in the vagina as demonstrated in Table 4. Similarly, around one third of them had to insert their fingers in the vagina to either start or complete emptying of the bladder or to empty the bowel (Table 4).

**Table 4:** Severity of vaginal soreness and the need to insert the finger into the vaginal to complete bladder or bowel emptying. The percentages are out of the 127 women who complaint of a dragging lump in the vagina

	Not at all	Occasionally	Sometimes	Most of times	All the times
Are you aware of soreness of your vagina?	27 %	50 %	13 %	5 %	5 %
Do you have to insert finger into your vagina to start or complete emptying your bladder?	66 %	19 %	9 %	3 %	3 %
Do you have to insert finger into your vagina to empty your bowel?	62 %	24 %	9 %	3 %	2 %

## Discussion

The findings of the present study indicated that symptoms of POP are highly prevalent among women in UAE. Prevalence of POP symptoms in our study appears to be higher than what was previously reported from the western countries [3–8]. The exact reason for this difference is difficult to ascertain without performing a comparative study; however, it could potentially reflect the difference in study populations and or risk factors. High prevalence of increased BMI, and high birth weight in our society may attribute to higher prevalence of POP in the current study [10]. Indeed, both these factors were found to be independent risk factor for having POP symptoms, similar to other studies some of which had used multivariate analysis [2, 9, 11].

In the present study, several factors were shown to be significantly associated with POP on univariate analysis. These findings were similar to other studies in which the data was analysed using univariate analysis only [1, 7, 11–13]. Age, however, was not found to be associated with POP in our population. This finding was in agreement with previous studies [11, 12].

In the current study, the nature of occupation was found to be significantly and independently associated with the prevalence of POP symptoms in such a way that women with physical jobs such as nurses, for instance, were associated with less POP symptoms. This could possibly be explained by the fact that these types of jobs would improve the pelvic musculature in women and hence decreases the pelvic organ descent. Women with other types of jobs or housewives in our population tend to do minimal exercise due to cultural reasons and the widespread employment of several housemaids per household which would leave women with minimal exercise to do unless they go to work or get involved in some form of gymnastic activities which is not common in this society.

In addition to the type of occupation, the level of education independently determined the prevalence of POP symptoms. Surprisingly, women with university degree had less prevalence of POP symptoms compared to women with lower level of education or illiterate women. This could be due to the fact that university graduates are more aware of healthy life style techniques including pelvic floor exercise compared to other women. Alternatively, these women tend to be more open in discussing their health issues. Certainly, more research is required to clarify this issue.

In the current study, we also investigated the relationship of POP symptoms with some underlining medical conditions. With this regard, both chronic chest disease and chronic constipation have been found to be independent risk factors for developing POP symptoms. Both conditions are associated with chronic straining and increased intra-abdominal pressure and this finding is consistent with previous reports [2, 11, 12]. So although constipation could be a consequence of POP, some studies has shown that constipation in the early age before the onset of POP was significantly more common in women who subsequently developed POP (61 %) compared to women who did not (4 %) [14].

To the best of our knowledge, this is the first study which assessed the prevalence of POP symptoms among women from this part of the world. One of the limitations of the study is the exclusion of nulliparous women younger than 30 years of age. This was based on the previously published data which showed a low incidence of POP in nulliparous women [7].

Due to the conservative nature of this society, it was difficult to carry out vaginal examination to identify women who had anatomical POP. However, a good correlation has been shown to exist between POP symptoms and the present of POP on vaginal examination [15]. In the current study, such symptoms were identified using a pre-tested questionnaire which included questions used in many previous studies [7, 12, 16, 17]. The use of healthcare providers to question the participants and fill the questionnaire eliminated the potential misunderstanding of the questions by the participants, which may have been interpreted differently otherwise.

In the current report, all women from the three family development centres who met the inclusion criteria were enrolled in the study. These centres are used for social, cultural and educational activities and are visited by women from different social and cultural backgrounds and hence more representative of the general population compared to subjects from hospitals or primary healthcare centres. The fact that the women included in the current study came from only one city makes it difficult to generalise the results to the whole country. However, the fact that this city (Al Ain) is the home for approximately 20 % of the whole national Emirati population [18, 19] indicates that the results obtained might provide a reasonable estimate of the prevalence of the POP in the whole country.

## Conclusion

In conclusion, symptoms of pelvic organ prolapse appear to be prevalent among Emirati women. History of chronic constipation and chest disease, level of education, job type, birth weight and body mass index were independent risk factors for having symptoms of pelvic organ prolapse. Additional healthcare programs and campaigns are required to educate the public regarding these risk factors to decrease the prevalence of this potentially bothersome condition.

## Additional file

Additional file 1:
**Pelvic organ prolapse questionnaire.**

## Abbreviations

UAE: United Arab Emirates
POP: Pelvic organ prolapse
BMI: Body mass index
