# Supplementary material for: Prevalence, risk factors and severity of symptoms of pelvic organ prolapse among Emirati women
Source: BMC Urol. 2015 Jul 7;15:66. doi: 10.1186/s12894-015-0062-1 (PMC4494174; doi:10.1186/s12894-015-0062-1)
Supplement: Additional file 1: — Pelvic organ prolapse questionnaire. [file 12894_2015_62_MOESM1_ESM.doc]

**Prevalence, risk factors and characters of symptoms of pelvic organ prolapse among Emirati women**

**Name:**

1. Age
2. Height (cm) 3. Weight (KG) 4. BMI

5**.** Occupation:

A. Housewife B. Secretarial job C. Job required heavy physical activities

6. Monthly income:

A. <5000 dirhams B. 5000-100000 dirhams C.> 100000 dirhams

7. Level of Education:

A. Illiterate B. Primary School C. High School D. University /higher education

8. Marital Status

A. Single B. Married C. Divorced D. Widow

**Obstetrics History**

9. Gravida 10. Para

11. Mode, number of delivery and birth weight:

**Mode Number Birth Weight (Maximum)(kg)**

1. Normal
2. Instrumental vaginal Delivery

(Vacuum or Forceps)

1. Emergency caesarean section
2. Elective Caesarean section

**Previous Medical History**

**Yes No**

12. History of urinary incontinence

13. History of previous surgery for urinary incontinence

14. History of previous surgery for vaginal prolapse

15. History of chronic chest problem (/chronic cough)

16. History of chronic constipation

17. History of Diabetes Mellitus

18. History of smoking

19. History of other diseases

If yes, what dieases:

**Questions about vaginal prolapse:**

**20.**  **Are you aware of dragging lump coming down in your vagina, lump coming out of your vagina or lump you can feel or see outside your vagina?**

A- Never

B-Occasionally

C-Sometimes

D-Most of the time

E-All the time

**21. Are you aware of soreness in your vagina?**

A- Never

B- Occasionally

C- Sometimes

D- Most of the time

E- All the time

**22. Do you have to insert finger into your vagina to start or complete emptying your bladder?**

A- Never

B- Occasionally

C- Sometimes

D- Most of the time

E- All the time

**23. Do you have to insert finger into your vagina to empty your bowels?**

A- Never

B- Occasionally

C- Sometimes

D- Most of the time

E- All the time
